# Editorial: Towards Real-World Deployment of Legged Robots

**DOI:** 10.3389/frobt.2021.829403

**Published:** 2022-03-03

**Authors:** Navinda Kottege, Luis Sentis, Dimitrios Kanoulas

**Affiliations:** ^1^ Commonwealth Scientific and Industrial Research Organisation (CSIRO), Pullenvale, QLD, Australia; ^2^ Department of Aerospace Engineering and Engineering Mechanics, University of Texas at Austin, Austin, TX, United States; ^3^ Department of Computer Science, University College London (UCL), London, United Kingdom

**Keywords:** legged robot, control, locomotion, actuation, exoskeleton

## 1 Introduction

Legged robots have unique potential advantages over wheeled and tracked systems in regard to the traversal of rough and unstructured terrain as well as rapid movement among crowds, and this has led to a growing interest and body of research in legged systems. However, actual real-world deployment of legged robots is still an emerging area. Furthermore, there has been no significant uptake of legged robots in domains such as agriculture, mining, manufacturing, environmental monitoring, and social navigation. Despite growing research interest in legged locomotion, a number of “missing ingredients” hold legged robots back from widespread real-world deployment. Some of the issues faced by researchers include the performance limitations of current legged robots (when compared to biological systems), the inherent lower efficiency of legged versus wheeled locomotion, design constraints related to available materials and actuators, substantial power requirements, and lack of spatial reasoning in constrained and dynamic environments; there are, however, many other challenges to be addressed. The goal of this special issue was to identify the fundamental research challenges whose solution is required to bring legged robots to real-world applications. This includes challenges in legged robot design, control, planning, perception, and system integration.

## 2 Overview of the Contents of the E-Book

### 2.1 BALLU2: A Safe and Affordable Buoyancy-Assisted Biped

Chae et al. present a new kind of bipedal robot, BALLU2, that uses helium balloons for buoyancy to lift part of its weight up while walking. This novel design provides the advantage of being extremely safe and low-cost. The design of the robot incorporates tendon wires to actuate the articulations attached to its thin linkages. The robot employs a data-driven neural network approach for performing walking behaviors. Employing such an approach enables handling of the underactuated nature of the robot as BALLU2 has only two actuated degrees of freedom per leg. In order to determine the transition function for walking, the authors perform a correlation study to choose significant state variables. Then, they train a neural network to learn transition times in both single and double support phases. Walking is accomplished in the real system at a speed of 0.18 m/s using an RGBD camera for onboard state estimation.

### 2.2 Terrain Perception-Free Quadrupedal Spinning Locomotion on Versatile Terrains: Modeling, Analysis, and Experimental Validation

Zhu et al. present a method for quadruped robots to perform spinning locomotion without the need for explicit terrain perception. While dynamic locomotion of quadrupedal robots over rough terrain is being addressed in the literature, spinning locomotion, akin to animals in nature, is rarely addressed. The presented work proposes a method for quadruped robots to perform spinning locomotion without explicit terrain sensing, including on sloped and stair terrain. The method also includes a controller to keep the spinning radius strictly bounded. The article presents results of real-world experimental validation of the proposed method on flat ground, slopes, and stairs.

### 2.3 An Efficient and Versatile Framework for Dynamic Motion Generation and Execution in Real-Life Settings: Application to Atalante Evolution With a Complete Paraplegic Person at the Cybathlon 2020 Exoskeleton Race

Huynh et al. present a framework for a dynamic trajectory generation method for a crutch-less lower limb exoskeleton Atalante Evolution from Wandercraft. This work allows a paraplegic user to select the modes of operation and actively cooperate to execute stable locomotion. The method was tested at the Cybathlon 2020 exoskeleton race where the pilot managed to successfully complete 4 out of 6 tasks. The authors present the details of the system as well as the Cybathlon competition where it was demonstrated to work in a real-life scenario. The results show how the method led to stable dynamic movements of the exoskeleton, hands-free walking, more natural stand-up and turning moves, and consequently a better physical condition of the pilot after the race compared to other competitors.

### 2.4 Autonomous Obstacle-Crossing Strategies for the Hybrid Wheeled-Legged Robot Centauro

De Luca et al. present a path planning system for hybrid wheeled-legged robots. Such robots that can roll around and step on/over obstacles are complex in controlling and decision-making. The authors have used LiDAR-based sensing to model the environment and decide on rolling or stepping on/down/over obstacles, to traverse challenging terrains with obstacles. Finally, the authors have tested the methodology in simulation and validated it on the real hybrid wheeled-legged Centauro robot in various scenarios.

### 2.5 Formulating and Deploying Strength Amplification Controllers for Lower-Body Walking Exoskeletons

Thomas et al. present a method for force amplification for human-directed tasks in exoskeletons. This work focuses on an amplification controller that augments human strength in a task agnostic manner with force torque sensors at the human–machine interface while being fully back-drivable. Evaluations of the proposed system are carried out with a powered lower-limb exoskeleton in a lab environment, where in addition to providing gravity compensation, the exoskeleton can compensate for unknown backpack payloads that are introduced during tasks such as walking around and stair climbing.

### 2.6 Versatile Locomotion Planning and Control for Humanoid Robots

Ahn et al. present the development of TOWR+, a motion planning framework, and IHWBC, a whole-body controller. Both are challenging problems for legged robots with a high number of degrees of freedom. The TOWR+ motion planner achieves arm motions for aiding in the robot’s inertia while walking, while IHWBC hierarchically handles tasks and soft constraints for unilateral contacts. Finally, the integrated TOWR+ and IHWBC lightweight and open-sourced framework was experimentally validated on the DRACO robot for zero-velocity steps, which is novel for bipeds with significant mass legs.

### 2.7 Omnidirectional Walking Pattern Generator Combining Virtual Constraints and Preview Control for Humanoid Robots

Ruscelli et al. present an omnidirectional walking pattern generator for bipedal locomotion. The work is based on biomechanics, i.e., when walking, humans tend to generate dynamic motion in the sagittal plane. The novelty of the methodology lies in the virtual constraints and online gait adaptation. Low computational complexity and high flexibility were the goals of the authors, and they were demonstrated on the COMAN+ humanoid robot for open-loop stable walking.

## 3 Conclusion

The seven articles in this e-book cover a broad range of legged robot applications in real-world scenarios. These span from robots with novel actuation methods for safe operation, exoskeletons for crutch-free locomotion for paraplegic persons, and novel controllers for humanoid robots to terrain perception methods for controller switching. Each article clearly presents the methods, provides results, and analyzes how these systems perform in the real world. These state-of-the-art methods address some of the missing ingredients needed for widespread deployment of legged robots in the real world. We believe this article collection will undoubtedly progress the exciting and active field of legged robotics, promoting more real-world deployments in the future.

